# Peripapillary microvasculature in patients with diabetes mellitus: An optical coherence tomography angiography study

**DOI:** 10.1038/s41598-019-52354-8

**Published:** 2019-11-01

**Authors:** Yong-Il Shin, Ki Yup Nam, Seong Eun Lee, Min-Woo Lee, Hyung-Bin Lim, Young-Joon Jo, Jung-Yeul Kim

**Affiliations:** 1 Department of Ophthalmology, Chungnam National University College of Medicine, Daejeon, Republic of Korea; 2 Department of Ophthalmology, Gyeongsang National University Changwon Hospital, Changwon, Republic of Korea

**Keywords:** Diabetes complications, Retinal diseases, Outcomes research

## Abstract

To evaluate changes in peripapillary microvascular parameters in diabetes mellitus (DM) patients using optical coherence tomography angiography (OCTA). Seventy-one diabetic patients (40 in the no diabetic retinopathy [DR] group and 31 in the non-proliferative DR [NPDR] group) and 50 control subjects. OCTA (Zeiss HD-OCT 5000 with AngioPlex) 6 × 6 mm scans centered on the optic disc were analyzed. Peripapillary vessel density (VD), perfusion density (PD) in superficial capillary plexus (SCP) were automatically calculated. The average macular ganglion cell-inner plexiform layer (mGC-IPL) and peripapillary retinal nerve fiber layer (pRNFL) thicknesses of the no DR and NPDR groups were significantly thinner than those of the control group. The no DR and NPDR groups showed lower peripapillary VD and PD in SCP compared with the control group. Using univariate regression analyses, the average mGC-IPL thickness, the pRNFL thickness, the no DR group and NPDR group were significant factors that affected the peripapillary VD and PD in SCP. Multivariate regression analyses showed that the grade of DR was a significant factor affecting the peripapillary VD and PD in SCP. OCTA revealed that peripapillary microvascular parameters in the no DR and NPDR groups were lower than those of normal controls. The peripapillary VD and PD in SCP were correlated with the mGC-IPL thickness, the pRNFL thickness, and the no DR and NPDR groups. Changes in peripapillary OCTA parameters may help with understanding the pathophysiology of DM and evaluating a potentially valuable biomarker for patients with subclinical DR.

## Introduction

Diabetic retinopathy (DR) is the most common retinal vascular disorder, and remains the leading cause of blindness in working-age adults worldwide^1,2^. Despite the significance of this problem, and the rising prevalence of diabetes, especially in emerging Asian countries^3^, the worldwide number of patients with DR will increase from 126.6 million in 2010 to 191.0 million by 2030^4^.

Diabetes mellitus (DM) is a complex metabolic disease that affects the microvascular system. It affects systemic tissues including the eyes, and is the most important and serious systemic diseases affecting eyes. DR is one of three important microvascular complications along with diabetic nephropathy and diabetic neuropathy.

Retinal diabetic neurodegeneration (RDN), characterized by inner retinal neurodegeneration, may precede the earlier stages of DR, or even occur when DR cannot be detected by ophthalmic examinations^5,6^. Some studies have reported a decrease in the macular ganglion cell-inner plexiform layer (mGC-IPL) and peripapillary retinal nerve fiber layer (pRNFL) thinning due to neuronal apoptosis and glial cell activation^7,8^. The mGC−IPL and pRNFL thinning can be easily measured and quantified by spectral domain–optical coherence tomography (SD-OCT). Recently, optical coherence tomography angiography (OCTA) has been used as a new and noninvasive imaging modality that allows microvascular visualization of the retina and choroid in various layers, as well as quantitative measurements of perfusion, in the optic nerve head, peripapillary and macular areas.

Recent studies using OCTA have shown that the vascular density of the macula is decreased in patients with DM without DR when compared with control subjects^9–11^. We hypothesized that peripapillary microvascular changes could be affected by the reduction of the pRNFL thickness by RDN. We therefore evaluated changes in peripapillary microvascular parameters among control subjects and diabetic patients with or without signs of DR using OCTA, and investigated the associations between peripapillary OCTA parameters and OCT parameters.

## Results

### Patient characteristics

A total of 71 diabetic patients (40 in the no DR group and 31 in the NPDR group) and 50 control subjects were enrolled. The mean ages of the control, no DR, and NPDR groups were 56.8 ± 9.1, 58.7 ± 9.5, and 59.0 ± 12.3 years, respectively, and no significant difference in age was found among the three groups. There were significant differences in the duration of diabetes and HbA1C between the no DR and NPDR groups (6.2 ± 6.4 vs 14.0 ± 9.5 years, *p* < 0.001; 6.82 ± 0.98 vs 8.03 ± 1.19%, *p* < 0.001, respectively). There was no significant difference among the three groups in sex, laterality, BCVA, SE, IOP, AL, and SS of OCTA images (Table 1).

**Table 1:** Demographics and clinical features of the study subjects.

	Control group (n = 50)	No DR group (n = 40)	NPDR group (n = 31)	*p*-value
Age (years)	56.8 ± 9.1	58.7 ± 9.5	59.0 ± 12.3	0.542^*^
Sex (male/female)	19/31	12/28	12/19	0.669^†^
Laterality (od/os)	25/25	19/21	15/16	0.971^†^
Duration of diabetes (years)		6.2 ± 6.4	14.0 ± 9.5	**<0.001** ^**‡**^
HbA1c (%)		6.82 ± 0.98	8.03 ± 1.19	**<0.001** ^**‡**^
BCVA (logMAR)	−0.01 ± 0.07	0.01 ± 0.10	0.02 ± 0.07	0.350^*^
Spherical equivalent (diopters)	−0.20 ± 0.97	−0.31 ± 1.93	−0.17 ± 1.56	0.917^*^
Intraocular pressure (mmHg)	15.1 ± 2.8	15.8 ± 2.8	15.9 ± 2.8	0.415^*^
Axial length (mm)	23.68 ± 0.96	23.67 ± 0.70	23.50 ± 0.71	0.567^*^
Signal strength	9.7 ± 0.5	9.7 ± 0.4	9.7 ± 0.5	0.993^*^

DR = diabetic retinopathy, NPDR = nonproliferative diabetic retinopathy; HbA1c = hemoglobin A1C; BCVA = best-corrected visual acuity; logMAR = logarithm of the minimum angle of resolution.

Values are presented as mean ± standard deviation unless otherwise indicated.

^*^p-value for one-way analysis of variance.

^†^p-value for chi-squared test.

^‡^p-value for Student’s *t*-test (no DR vs. NPDR group).

### OCT measurements

The CMT did not show a statistically significant difference among the three groups (*p* = 0.234). The average mGC-IPL thicknesses of the no DR (81.4 ± 4.8 µm) and NPDR (80.0 ± 6.5 µm) groups were significantly thinner than those of the control group (85.3 ± 4.8 µm, *p* < 0.001). The average pRNFL thicknesses of the no DR (92.8 ± 5.9 µm) and NPDR (90.0 ± 8.2 µm) groups were also significantly thinner than those of the control group (97.5 ± 8.2 µm, *p* < 0.001). The pRNFL thickness of the inferior quadrant was significantly thinner in the no DR and NPDR groups than those of the control group (*p* = 0.001) (Table 2).

**Table 2:** Comparison of the central macular thickness, average mGC-IPL thickness, and pRNFL thickness among groups.

	Control group (n = 50)	No DR group (n = 40)	NPDR group (n = 31)	*p*-value^*^ (post-hoc)
Central macular thickness (μm)	252.7 ± 19.8	249.5 ± 22.0	244.6 ± 19.9	0.234
Average mGC-IPL thickness (μm)	85.3 ± 4.8	81.4 ± 4.8	80.0 ± 6.5	**<0.001** (control >No DR,NPDR)
pRNFL thickness (μm)				
Average	97.5 ± 8.2	92.8 ± 5.9	90.0 ± 8.2	**<0.001** (control >No DR,NPDR)
Superior	120.5 ± 13.7	115.7 ± 11.8	114.3 ± 13.5	0.078
Nasal	70.2 ± 8.7	68.3 ± 11.2	65.2 ± 10.7	0.052
Inferior	125.8 ± 14.4	117.4 ± 13.0	114.0 ± 16.0	**0.001** (control >No DR,NPDR)
Temporal	73.2 ± 10.5	71.4 ± 8.6	68.0 ± 12.5	0.100

mGC-IPL = macular ganglion cell-inner plexiform layer; pRNFL = peripapillary retinal nerve fiber layer; DR = diabetic retinopathy, NPDR = nonproliferative diabetic retinopathy.

Values are presented as mean ± standard deviation.

^*^p-value for one-way analysis of variance.

### Peripapillary OCTA measurements

The mean values ± standard deviation of OCTA measurements among the three groups are presented in Table 3 (VD) and Table 4 (PD). The average peripapillary VD and PD in superficial capillary plexus (SCP) of the inner ring, outer ring, and peripapillary area were significantly different among the three groups (all, *p* < 0.05). The no DR and NPDR groups showed a lower peripapillary VD and PD compared with the control group. The peripapillary VD of all sectors of the outer ring were lower in the NPDR group compared with the control group. The temporal quadrant of the inner ring and the nasal, inferior, temporal quadrants of the outer ring showed a lower peripapillary PD compared with the control group.

**Table 3:** Comparison of superficial peripapillary vessel density among groups.

	Control group (n = 50)	No DR group (n = 40)	NPDR group (n = 31)	*p*-value^*^	*p*-value^†^	*p*-value^‡^	*p*-value^§^
Peripapillary area	18.76 ± 0.58	18.32 ± 0.48	17.91 ± 0.82	**<**0.001	0.004	**<**0.001	0.020
Inner ring							
Average	18.06 ± 0.98	17.37 ± 1.00	17.27 ± 1.19	0.001	0.006	0.003	1.000
Superior	18.14 ± 1.16	17.47 ± 1.37	17.42 ± 1.49	0.022	0.057	0.060	1.000
Nasal	18.35 ± 1.11	17.94 ± 1.32	17.86 ± 1.24	0.139			
Inferior	18.50 ± 0.79	18.18 ± 0.77	18.15 ± 1.11	0.126			
Temporal	16.84 ± 2.24	15.87 ± 1.98	15.63 ± 2.57	0.035	0.131	0.062	1.000
Outer ring							
Average	18.96 ± 0.68	18.61 ± 0.60	18.10 ± 0.93	**<**0.001	0.049	**<**0.001	0.014
Superior	19.19 ± 0.60	18.92 ± 0.74	18.80 ± 0.82	0.042	0.231	0.049	1.000
Nasal	17.55 ± 1.30	17.00 ± 1.14	16.77 ± 1.41	0.020	0.138	0.027	1.000
Inferior	19.06 ± 0.81	18.82 ± 1.09	18.17 ± 1.38	0.002	0.904	0.001	0.038
Temporal	19.94 ± 1.10	19.41 ± 1.29	18.69 ± 1.63	**<**0.001	0.177	**<**0.001	0.072

DR = diabetic retinopathy, NPDR = nonproliferative diabetic retinopathy.

Values are presented as mean ± standard deviation.

^*^The p-value was obtained using one-way analysis of variance.

^†^The p-value was obtained using post hoc tests (Bonferroni) between the normal control group and the no DR group.

^‡^The p-value was obtained using post hoc tests (Bonferroni) between the normal control group and the NPDR group.

^§^The p-value was obtained using post hoc tests (Bonferroni) between no DR group and NPDR group.

**Table 4:** Comparison of superficial peripapillary perfusion density among groups.

	Control group (n = 50)	No DR group (n = 40)	NPDR group (n = 31)	*p*-value^*^	*p*-value^†^	*p*-value^‡^	*p*-value^§^
Peripapillary area	0.479 ± 0.012	0.469 ± 0.018	0.459 ± 0.021	**<**0.001	0.007	**<**0.001	0.036
Inner ring							
Average	0.473 ± 0.026	0.460 ± 0.027	0.456 ± 0.027	0.009	0.049	0.016	1.000
Superior	0.490 ± 0.027	0.481 ± 0.039	0.481 ± 0.029	0.336			
Nasal	0.491 ± 0.028	0.481 ± 0.037	0.474 ± 0.034	0.058			
Inferior	0.493 ± 0.029	0.492 ± 0.023	0.487 ± 0.033	0.618			
Temporal	0.418 ± 0.052	0.384 ± 0.051	0.379 ± 0.057	0.003	0.014	0.008	1.000
Outer ring							
Average	0.481 ± 0.013	0.471 ± 0.021	0.459 ± 0.025	**<**0.001	0.049	**<**0.001	0.039
Superior	0.498 ± 0.015	0.493 ± 0.022	0.489 ± 0.021	0.127			
Nasal	0.450 ± 0.035	0.437 ± 0.032	0.425 ± 0.041	0.011	0.314	0.009	0.449
Inferior	0.492 ± 0.017	0.482 ± 0.030	0.468 ± 0.033	**<**0.001	0.183	**<**0.001	0.097
Temporal	0.487 ± 0.016	0.472 ± 0.033	0.455 ± 0.047	**<**0.001	0.125	**<**0.001	0.079

DR = diabetic retinopathy, NPDR = nonproliferative diabetic retinopathy.

Values are presented as mean ± standard deviation.

*The p-value was obtained using one-way analysis of variance.

^†^The p-value was obtained using post hoc tests (Bonferroni) between the normal control group and the no DR group.

^‡^The p-value was obtained using post hoc tests (Bonferroni) between the normal control group and the NPDR group.

^§^The p-value was obtained using post hoc tests (Bonferroni) between no DR group and NPDR group.

**Figure 1:**
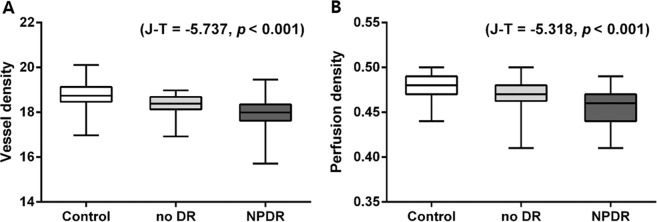
The tendency of average peripapillary area vessel density and perfusion density among groups. Boxes are 25% to 75% (lower to upper) quartiles, lines in boxes are medians, and whiskers indicate variability (minimum and maximum values). The Jonckheere–Terpstra test showed significant negative trends in the order of the control, no DR, and NPDR groups of the peripapillary area (**A**) vessel density and (**B**) perfusion density (Jonckheere-Terpstra [J-T] statistic = −5.737, *p* < 0.001; J-T statistic = −5.318, *p* < 0.001, respectively).

The Jonckheere–Terpstra test showed significant negative trends in the order of the control, no DR, and NPDR groups of the peripapillary area VD and PD (Jonckheere-Terpstra [J-T] statistic = −5.737, *p* < 0.001; J-T statistic = −5.318, *p* < 0.001, respectively) (Fig. 1).

### Association of clinical and peripapillary OCTA parameters with pRNFL thickness

**Figure 2:**
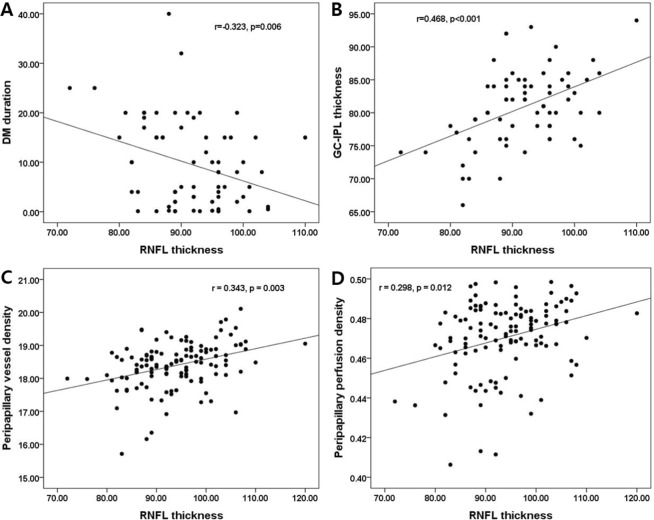
Scatter plots of association with peripapillary retinal nerve fiber layer (pRNFL) thickness in diabetic patients. The duration of diabetes, average macular ganglion cell-inner plexiform layer (mGC-IPL) thickness, peripapillary area vessel density (VD) and perfusion density (PD) in superficial capillary plexus were significantly correlated with pRNFL thicknesses. Correlation coefficients (r) and *p-*values are shown.

In diabetic patients, the duration of diabetes (r = −0.323, *p* = 0.006), average mGC-IPL thickness (r = 0.468, *p* < 0.001), peripapillary VD (r = 0.343, *p* = 0.003), and PD (r = 0.298, *p* = 0.012) in the peripapillary areas were significantly correlated with pRNFL thicknesses. The average mGC-IPL thickness, and peripapillary VD and PD were positively correlated, and the duration of diabetes was negatively correlated with pRNFL thicknesses (Fig. 2). However, age, female sex, BCVA, SE, IOP, AL, and CMT were not correlated with the average peripapillary VD and PD. In the control group, the pRNFL thickness was not correlated with clinical and peripapillary OCTA parameters.

### Linear regression analysis for determine factors associated with peripapillary OCTA parameters

Using univariate regression analysis, the average mGC-IPL thickness (β = 0.040, *p* < 0.001) and average pRNFL thickness (β = 0.032, *p* < 0.001) of the no DR (β = −0.434, *p* < 0.001) and NPDR groups (β = −0.847, *p* < 0.001) were significant factors, which affected the peripapillary VD. Multivariate regression analysis showed that the no DR and NPDR groups were significant factors affecting the peripapillary VD (no DR: β = −0.325, *p* = 0.020; NPDR: β = −0.681, *p* < 0.001) (Table 5).

**Table 5:** Univariate and multivariate regression for determining the factors associated with peripapillary area vessel density.

	Univariate analysis		Multivariate analysis	
	β (95% CI)	*p*-value	β (95% CI)	*p*-value
Age (years)	−0.012 (−0.024, 0.001)	0.060		
Female sex	−0.147 (−0.412, 0.119)	0.277		
No DR vs. control	−0.434 (−0.661, −0.207)	**<0.001**	−0.325 (−0.598, −0.053)	**0.020**
NPDR vs. control	−0.847 (−1.158, −0.535)	**<0.001**	−0.681 (−0.988, −0.375)	**<0.001**
BCVA (LogMAR)	−1.361 (−2.934, 0.213)	0.089		
Spherical equivalent (diopter)	−0.010 (−0.096, 0.077)	0.823		
Intraocular pressure (mmHg)	−0.007 (−0.053, 0.038)	0.745		
Axial length (mm)	0.022 (−0.136, 0.179)	0.783		
Central macular thickness (μm)	0.003 (−0.003, 0.010)	0.273		
Average mGC-IPL thickness (μm)	0.040 (0.019, 0.062)	**<0.001**	0.010 (−0.014, 0.034)	0.402
Average pRNFL thickness (μm)	0.032 (0.017, 0.047)	**<0.001**	0.015 (−0.002, 0.032)	0.087

β = regression coefficients; CI = confidential interval; DR = diabetic retinopathy; NPDR = nonproliferative diabetic retinopathy; BCVA = best-corrected visual acuity; LogMAR = logarithm of the minimum angle of resolution; mGC-IPL = macular ganglion cell-inner plexiform layer; pRNFL = peripapillary retinal nerve fiber layer.

Using univariate regression analysis, the average mGC-IPL thickness (β = 0.001, *p* = 0.004) and average pRNFL thickness (β = 0.001, *p* = 0.001) of the no DR (β = −0.011, *p* = 0.001) and NPDR groups (β = −0.021, *p* < 0.001) were significant factors, which affected the peripapillary PD. Multivariate regression analysis showed that the no DR and NPDR groups were significant factors affecting the peripapillary PD (no DR: β = −0.009, *p* = 0.015; NPDR: β = −0.018, *p* < 0.001) (Table 6).

**Table 6:** Univariate and multivariate regression for determining the factors associated with peripapillary area perfusion density.

	Univariate analysis		Multivariate analysis	
	β (95% CI)	*p*-value	β (95% CI)	*p*-value
Age (years)	0.000 (0.000, 0.000)	0.613		
Female sex	−0.004 (−0.011, 0.003)	0.236		
No DR vs. control	−0.011 (−0.017, −0.005)	**0.001**	−0.009 (−0.016, −0.002)	**0.015**
NPDR vs. control	−0.021 (−0.028, −0.014)	**<0.001**	−0.018 (−0.026, −0.010)	<**0.001**
BCVA (LogMAR)	−0.022 (−0.063, 0.019)	0.298		
Spherical equivalent (diopter)	0.001 (−0.001, 0.004)	0.212		
Intraocular pressure (mmHg)	0.000 (−0.001, 0.001)	0.969		
Axial length (mm)	−0.001 (−0.005, 0.003)	0.579		
Central macular thickness (μm)	0.000 (0.000, 0.000)	0.589		
Average mGC-IPL thickness (μm)	0.001 (0.000, 0.001)	**0.004**	0.000 (−0.001, 0.001)	0.744
Average pRNFL thickness (μm)	0.001 (0.000, 0.001)	**0.001**	0.000 (0.000, 0.001)	0.188

β = regression coefficients; CI = confidential interval; DR = diabetic retinopathy; NPDR = nonproliferative diabetic retinopathy; BCVA = best-corrected visual acuity; LogMAR = logarithm of the minimum angle of resolution; mGC-IPL = macular ganglion cell-inner plexiform layer; pRNFL = peripapillary retinal nerve fiber layer.

## Discussion

We studied peripapillary microvascular changes in diabetic patients using OCTA. Our study showed mGC-IPL and pRNFL thinning in patients with diabetes compared with normal control subjects. The peripapillary VD and PD in SCP were also lower in the no DR and NPDR groups than in the control group. The average pRNFL and mGC-IPL thicknesses, in the no DR and NPDR groups were also correlated with both peripapillary VD and PD in SCP.

Early changes in DR occur through the loss of endothelial cells or pericytes in small vessels^12,13^. However, DR is clinically diagnosed by observing microaneurysm using funduscopy. Preclinical DR may therefore be accompanied by additional vascular abnormalities of the capillaries before the occurrence of microaneurysm. RDN may occur clinically before retinal microvascular abnormality is observed. These changes are accompanied by neural apoptosis and ganglion cell loss, which can be observed as thinning of the inner retina layer, including the mGC-IPL and pRNFL, using SD-OCT^8,14^. In our study, mGC-IPL and pRNFL thinning were observed in the no DR and NPDR groups, when compared with control subjects, which was consistent with previous studies. Therefore, our findings in diabetic patients with no DR may reflect the discrepancies between the clinical DR grading and changes of anatomical structures of the inner retina, as shown by OCT.

Microvascular perfusion of the optic disc can be studied using recently developed OCTA. The peripapillary perfusion in glaucoma patients is reduced, suggesting that the optic nerve head (ONH) vascular dysfunction may be associated with glaucoma pathogenesis^15,16^. Blood flow of the ONH is supplied by the posterior ciliary artery and central retinal artery. The radial peripapillary capillary (RPC) is the most superficial layer of the capillary network, supplying blood to the superficial pRNFL around the ONH. Evaluation of the RPC has been limited because of the difficulties of imaging these vessels using conventional fluorescein angiography. However, the development of OCTA has facilitated visualization and quantification of the RPC.

There have been some recent reports of peripapillary microvascular changes in DM patients^17–19^. They reported that the RPC density and the pRNFL thickness were significantly decreased in subclinical DR patients when compared with control subjects. There are no established methods of determining the appropriate area for measuring peripapillary microvascular perfusion. These studies used RPC density to measure the peripapillary microvascular perfusion areas in circles with diameters of 3.45–4.5 mm, centered on the disc and 2-mm-diameter circle area centered on the disc was subtracted. Because there was a difference in disc size among individual patients^17,18^, ONH perfusion might be underestimated. We measured the peripapillary VD and PD in SCP automatically using software, which included both the RPCs and the large retinal vessels around the disc. Thus, it was a mixture of both disc and retinal circulation and not a pure measurement of a single vascular bed. This measurement may be more relevant because it evaluated both the optic nerve and the retinal blood-flow supply in the peripapillary area. Jia *et al*.^20^ found that relatively dense RPC extends as far as 5.5 mm from the disc center, and large area measurement is better for examining the changes in the RPC^21^. so we measured 6 × 6 mm scan area centered on the optic disc, which was wider than that of previous studies.

In our study, patients with both DM and systemic HTN were excluded. Previous studies reported that patients with HTN have decreased macular OCTA parameters compared with controls^22,23^. It may also affect the measurement of the peripapillary OCTA parameters^24^. Vujosevic *et al*.^19^ showed differences in baseline systemic blood pressure between groups. In a study by Rodriques *et al*.^18^, 83% of the patients had HTN (especially among moderate NPDR: 94.8%), which may have affected the measured values. We tried to determine only the changes of peripapillary microvascular perfusion caused by diabetes, excluding the effect of HTN.

In our study, the VD and PD values of the peripapillary areas and average inner rings and outer rings in diabetic patients were significantly different from those of control subjects. Previous studies also showed a decrease in RPC density compared with the control group and the no DR group, which was consistent with the results of our study^17–19^. As the DR severity progressed, the VD and PD of the peripapillary areas and the average of the outer ring had a tendency to decrease, which represented a difference from previous reports. Rodriques *et al*.^18^ reported that there was no difference in annular RPC density according to the severity of DR. Vujosevic *et al*.^19^. reported that the peripapillary VD in the DR group was significantly smaller than that of the no DR group, but the peripapillary PD did not show a significant difference. Our study analyzed the segmentation of SCP including RPC, but Rodrigues^18^ and Vujosevic^19^ analyzed RPC only. This difference in segmentation might be the reason for the discrepancies in our results compared with previous studies. And The OCTA image scan size, instrument, and the area of the ONH perfusion boundary differed from previous studies. The HbA1c and duration of diabetes in the no DR and NPDR groups were also different from previous studies. The duration of diabetes in our NPDR group was long, and HbA1C was high compared with previous studies, so these may be more affected, resulting in differences between the no DR and NPDR groups.

Compared with normal controls, the pRNFL thickness in diabetes patients was reduced^25,26^, and DM is a known risk factor for primary open-angle glaucoma^27–29^. In our study, the average mGC-IPL thickness, duration of diabetes, and peripapillary VD and PD in SCP were correlated with the pRNFL thickness in diabetic patients. This may be associated with progressive ganglion cells and astrocyte loss due to diabetes, and the long duration of DM would be more likely to show these results.

Using univariate analysis, the peripapillary VD and PD were correlated with the average mGC-IPL and the pRNFL thickness. The pRNFL contains axons of the RGCs that travel from RGC bodies to the optic disc, and the mGC-IPL consists of nuclei and dendrites of the RGCs. This suggests that reduced mGC-IPL and pRNFL thicknesses may be related to peripapillary retinal microcirculation. Multivariate regression analysis showed that the NPDR group was most related to the peripapillary VD and PD, and the no DR group was also related to the peripapillary VD and PD. Regression analysis can only know whether there is an association but not causalities.

This study had some limitations. First, the method of vessel density measurement was different in our study compared with those used in previous studies. We used the vessel density in SCP automatically provided by the software. This software did not differentiate between capillaries and large vessels. However, there is no established method to evaluate the only RPC. Further advances in technology may help to evaluate the capillaries and large vessels separately. Second, smoking habits/history could not be analyzed because it was often absent in medical records. Further study should be needed the effects of smoking on OCTA parameters. Finally, because of the cross-sectional study, it would be difficult to establish cause and effect between pRNFL thinning and decreased peripapillary perfusion. Prospective longitudinal studies should be performed to investigate this temporal relationship.

Our study have several strengths. First, we identified the factors associated with peripapillary microvasculature. Second, we enrolled DM patients without systemic HTN to exclude the additional effects of HTN. Third, we only analyzed good quality OCTA images with SS 9 or higher by excluding low vision, macular edema, and proliferative DR patients, which may have affected the measurement values. Additionally, we measured 6 × 6 mm scan area which was wider than that of previous studies to better understand the changes in peripapillary microvasculature.

In conclusion, peripapillary microvascular parameters in the no DR and NPDR groups were lower than those of normal controls using OCTA. These changes were correlated with the mGC-IPL thickness, the pRNFL thickness, and the no DR and NPDR groups. Changes in peripapillary OCTA parameters may help with understanding the pathophysiology of DM and evaluating a potentially valuable biomarker for patients with subclinical DR.

## Methods

### Study design

This was a cross-sectional, observational study. We reviewed the medical records of diabetic patients who visited the Retina Clinic of Chungnam National University Hospital for checkups for DR from April 2017 to November 2018. The study protocol was approved by the Institutional Review Board of Chungnam National University Hospital. The requirement for obtaining informed patient consent was waived due to the retrospective nature of the study. The study adhered to the tenets of the Declaration of Helsinki.

### Participants

Included patients were older than 18 years and diagnosed with type 2 DM, with or without DR (diagnosis was made using a dilated fundus examination and fundus photography, by JYK). The diabetic patients were divided into two groups: a no DR group and a non-proliferative DR (NPDR) group. The NPDR group consisted only of mild to moderate NPDR patients. DR grading was performed according to the International Clinical Classification of Diabetic Retinopathy disease severity scale^30^. Patients with severe NPDR, proliferative DR or diabetic macular edema were excluded. One eye from each participant was included in this study. A single eye was randomly selected if both eyes were eligible.

The exclusion criteria were as follows: (1) a history of retinal or optic nerve diseases or glaucoma; (2) a best-corrected visual acuity (BCVA) < 20/25; (3) a medical history of systemic hypertension (HTN); (4) high myopia (spherical equivalent [SE] > −6 diopters, axial length [AL] ≥ 26.0 mm); (5) previous treatment (laser photocoagulation, intravitreal injections, or vitrectomy); and (6) significant media opacity.

Normal control subjects without diabetes and with no ophthalmic disease were enrolled as the control group. Among the subjects who visited our clinic for various reasons (health screening checkup, work up for ocular disease, etc.), those who met inclusion and exclusion criteria were enrolled in the study. These patients wanted a comprehensive examination of the cataract, glaucoma, refractive error, other retinal abnormality, etc. In these patients, we performed a comprehensive ophthalmic examinations including OCT, OCTA, and AL.

All patients underwent measurement of their BCVA using a Snellen chart, intraocular pressure (IOP), SE and AL using an IOL master, version 5.0 (Carl Zeiss Meditec, Jena, Germany), slit-lamp biomicroscopy, a dilated fundus examination, SD-OCT, and OCTA.

### Spectral domain-optical coherence tomography measurement

SD-OCT examination was performed using Cirrus HD-OCT (Carl Zeiss Meditec, Dublin, CA, USA). The same one experienced examiner performed all imaging. Central macular thickness (CMT) and mGC-IPL thickness were measured using a macular cube 512 × 128 protocol. The ganglion cell analysis algorithm automatically measured mGC-IPL thickness by identifying the outer boundaries of the RNFL and IPL from the macular cube scan. The average, minimum, and sectoral (superotemporal, superior, superonasal, inferonasal, inferior, inferotemporal) thicknesses of the mGC-IPL are measured in an elliptical annulus (dimensions: vertical inner and outer radius of 0.5 mm and 2.0 mm, horizontal inner and outer radius of 0.6 and 2.4 mm, respectively) around the fovea^31^. Among the values measured by macular scan, CMT and average mGC-IPL were used for analysis. An optic disc cube 200 × 200 protocol was used for pRNFL measurements. This scan a 6 × 6 mm area centered on the disc. and the pRNFL thickness was measured from a circle of diameter 3.46 mm from the center of the optic disc^32^. The average and four-quadrant sector (superior, nasal, inferior, and temporal) thickness were used for pRNFL analysis. We excluded the signal strength (SS) less than 7 or segmentation error, or poor centration. Two images were taken of each patients, of which the greater SS image was chosen.

### Optical coherence tomography angiography measurement

OCTA examination was performed using the Zeiss Cirrus HD-OCT 5000 with AngioPlex, with a wavelength of 840 nm and an A-scan rate of 68KHz. The volumetric scans were processed using optical microangiography (OMAG) algorithms to generate the flow images. The OMAG algorithm analyzes differences in both phase and intensity information from repeated B-scans to quantify motion contrast. The superficial capillary plexus (SCP) was extended from the internal limiting membrane (ILM) to the IPL^33^.

**Figure 3:**
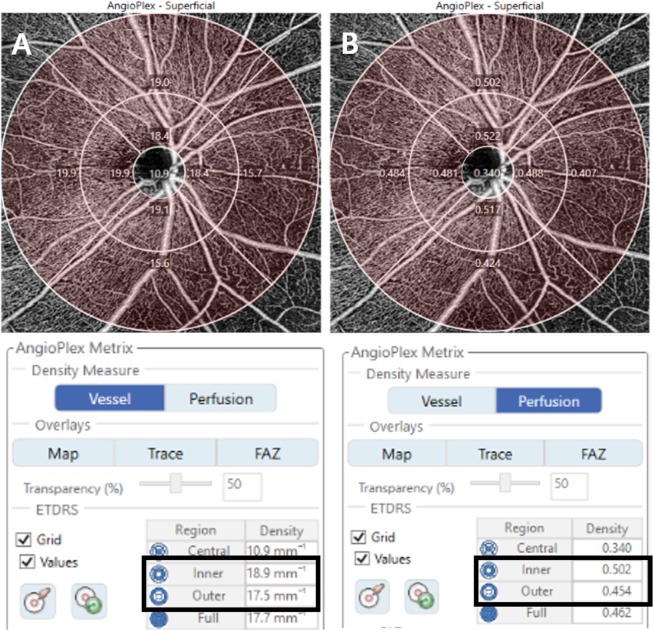
Optical coherence tomography angiography 6 × 6 mm scan image centered on the optic disc. The en face image of the superficial layer overlaid with the Early Treatment of Diabetic Retinopathy Study grid. The diameters of the three concentric circles are 1, 3, and 6 mm. The measurement tool (AngioPlex software, version 10.0; Carl Zeiss Meditec) provided (**A**) peripapillary vessel density and (**B**) perfusion density measurements in individual subfields. The bold box shows the automatic quantitative measurements for an average of the inner ring and outer ring. Area marked in pink shows the peripapillary area we defined.

The optic disc centered 6 × 6 mm scan were taken to investigate peripapillary microvasculature. A 6 × 6 mm scan were acquired containing 350 A-scans in each B-scan, and each B-scan is repeated twice at the same location. The vessel density (VD) defined as the total length of perfused vasculature per unit area in a region of measurement, and perfusion density (PD) defined as the total area of perfused vasculature per unit area in a region of measurement. Cirrus OCTA software (ver. 10.0) calculated automatically the value of the SCP according to the Early Treatment of Diabetic Retinopathy Study subfields^33^. The diameters of the three circles were 1, 3, and 6 mm, and each ring was divided into four quadrants. We analyzed the peripapillary VD and PD in SCP of the quadrants of the inner and outer rings, and average of the inner ring and outer ring using a previously described method^34^. We further analyzed the peripapillary area. Peripapillary area was defined as the full area except the central subfield (Fig. 3).

Lim *et al*.^35^ reported that OCTA measurements varied with the SS, and at least 9 is recommended. So, We included only images with SS ≥ 9 and without segmentation errors and motion artifacts. Two images were taken of each patients, of which the greater SS image was chosen.

### Statistical analysis

All statistical analyses were performed using SPSS statistical software for Windows, version 22.0 (SPSS, Chicago, IL, USA). For statistical analyses, BCVA values were transformed to the logarithm of the minimum angle of resolution (log MAR) values. One-way analysis of variance with Bonferroni correction and the chi-squared test were used to compare clinical factors, OCT parameters, and OCTA parameters among groups. Ordered differences among groups were analyzed by the Jonckheere–Terpstra (J-T) test for trends. Pearson’s correlation was used to investigate the associations between clinical, peripapillary OCTA parameters and pRNFL thicknesses. Univariate and multivariate regression analyses were also performed to identify factors significantly related to peripapillary VD and PD in SCP. A value of *p* < 0.05 was considered statistically significant.

## Data Availability

Data supporting the findings of the current study are available from the corresponding author on reasonable request.
